# Saccharine

**Published:** 1886-10

**Authors:** 


### Editorial.

### *SACCHARINE.*

This is a substance which is derived from coal-tar, and which undoubtedly will be of great use in medicine. It is named saccharine, from its very sweet taste. It is an aromatic hydrocarbon. The scientific name for saccharine is anhydrous orthosulphamine benzoic acid. It is a white powder composed of irregular crystals and little soluble in cold but quite so in hot water. Alcohol and ether dissolve it easily. Syrups of grape or starch-sugar also dissolve saccharine when warm, and they may thus be sweetened to almost any degree. Glycerine and other bodies may also be used as solvents. It crystallizes in short thick prisms, but the crystals are generally very small and badly defined. It melts at about 200° C. and partially decomposes at that temperature. Its very sweet taste can best be appreciated by tasting the smallest particle conceivable, but it can be somewhat understood if we say it is 300 times sweeter than cane sugar. In solution the sweet taste can be detected, if there be but 1 part to 70.000 of water, while cane sugar cannot easily be detected if it be dissolved in proportion of 1 to 300 parts of water. It is a powerful antiseptic and hence is indicated and harmless in many cases where sugar in any form would be harmful. It is a great boon then to patients who suffer from diabetes and to those corpulent persons who cannot forego the sweet flavor to their food-stuffs. It will greatly facilitate the carrying out of the strict diet so essential to diabetic patients but which it is almost an impossibility to follow. "As its solution forms an acid, which readily forms salts with bases, it can be utilized to combine directly with alkaloidal quinine." By this combination we have a quinine sulphamine-benzoate which has a sweet taste and is very pleasant to take. With morphia too we have the same combination and the same sweet substance.

"In fermentative disturbances of the digestive tract, when sugar and carbo-hydrates are contraindicated, it may be used as a corrective for the nauseant taste of other remedies, especially so as it has decided antiseptic powers. It may also be used as a condiment for milk and other nutriments to be taken. To improve the taste of elixirs, wines, etc., it seems especially fitted, as they will, under such conditions, have the very desirable property of not deranging the stomach. At the request of Dr. C. Fahlberg, its discoverer, many experiments were made by Drs. Victor Aducco and Dr. Hugo Mosso, of Turin, on animals, on themselves and patients. It was found that this substance does not undergo any change in the system, but is rapidly eliminated by the kidneys. Saccharine appeared in the urine in about a half hour, and at the end of twenty-four hours no more was discovered. They both took and administered this medicine to others, in minute and large doses, and observed no deleterious result. To several nursing women large doses were given, and there was no appearance of the substance in the milk. The sputas were also examined and there was also none of the substance present. It was determined that it was wholly eliminated by the kidneys, and, as has been before remarked, absolutely unchanged.

Patients taking this substance did not lose in flesh, even when as large doses as a drachm was given daily. It did not produce any abnormal effect or disturb any of the functions of the body. The appetite was increased in all the cases, and in some it was noticed that thirst was allayed. It is our duty then to give this a trial in diabetes, for the two great indications are thereby met; namely, exclusion of all sugar from the diet and the allaying of the intense diabetic thirst. At present it is only manufactured in Germany, where the products for its manufacture can best be obtained. It is doubtful if it will be manufactured here, at present, as the discoverer of the process has patented it in all countries. The combination, also, with the various alkaloids has been patented, hence, its usefulness in medicine is somewhat impaired. It may be interesting to note that saccharine may be used for sweetening grape or starch sugar, one to two parts of saccharine, mixed with 1,000 parts of starch-sugar, forming a substitute for cane-sugar where body and sweetness are required; for instance, this mixture is well adapted for manufacture of confectionery and liquors in the place of cane-sugar.

